# Analog/RF Performance of T-Shape Gate Dual-Source Tunnel Field-Effect Transistor

**DOI:** 10.1186/s11671-018-2723-y

**Published:** 2018-10-12

**Authors:** Shupeng Chen, Hongxia Liu, Shulong Wang, Wei Li, Xing Wang, Lu Zhao

**Affiliations:** School of Microelectronics, Key Laboratory of Wide Band-Gap Semiconductor Materials and Devices of Education, Xidian University, Xi’an, 710071 China

**Keywords:** T-shaped gate, Recessed gate, Tunnel field-effect transistor (TFET), Analog/RF performance

## Abstract

In this paper, a silicon-based T-shape gate dual-source tunnel field-effect transistor (TGTFET) is proposed and investigated by TCAD simulation. As a contrastive study, the structure, characteristic, and analog/RF performance of TGTFET, LTFET, and UTFET are discussed. The gate overlap introduced by T-shape gate can enhance the efficiency of tunneling junction. The dual-source regions in TGTFET can increase the on-state current (*I*_ON_) by offering a doubled tunneling junction area. In order to further improve the device performance, the n+ pocket is introduced in TGTFET to further increase the band-to-band tunneling rate. Simulation results reveal that the TGTFET’s *I*_ON_ and switching ratio (*I*_ON_/*I*_OFF_) reach 81 μA/μm and 6.7 × 10^10^ at 1 V gate to source voltage (*V*_g_). The average subthreshold swing of TGTFET (SS_avg_, from 0 to 0.5 V *V*_g_) reaches 51.5 mV/dec, and the minimum subthreshold swing of TGTFET (SS_min_, at 0.1 V *V*_g_) reaches 24.4 mV/dec. Moreover, it is found that TGTFET have strong robustness on drain-induced barrier lowering (DIBL) effect. The effects of doping concentration, geometric dimension, and applied voltage on device performance are investigated in order to create the TGTFET design guideline. Furthermore, the transconductance (*g*_m_), output conductance (*g*_ds_), gate to source capacitance (*C*_gs_), gate to drain capacitance (*C*_gd_), cut-off frequency (*f*_T_), and gain bandwidth (GBW) of TGTFET reach 232 μS/μm, 214 μS/μm, 0.7 fF/μm, 3.7 fF/μm, 11.9 GHz, and 2.3 GHz at 0.5 V drain to source voltage (*V*_d_), respectively. Benefiting from the structural advantage, TGTFET obtains better DC/AC characteristics compared to UTFET and LTFET. In conclusion, the considerable good performance makes TGTFET turn into a very attractive choice for the next generation of low-power and analog/RF applications.

## Background

The scaling down of metal-oxide-semiconductor field-effect transistors (MOSFETs) brings significant improvement in integrated circuit (IC) power consumption, switching characteristic, circuit function, and IC density [1, 2]. But the irreconcilable contradiction between the scaling of the supply voltage and the reduction of the off-state leakage currents (*I*_OFF_) will finally result in the unacceptable high power consumption [3]. At the same time, reliability degradation caused by short-channel effects (SCEs) becomes more and more serious [4, 5]. In order to address these problems, it is valid to reduce subthreshold swing (SS) and supply voltage of the devices. Based on the band-to-band tunneling mechanism, tunnel field-effect transistors (TFETs) reach the subthreshold swing (SS) smaller than 60 mV/dec and could effectively reduce the supply voltage [6–10]. Moreover, due to the existence of the tunneling junction near the source, TFET usually has a small gate to source capacitance (*C*_gs_) [1, 11] which is beneficial to the device frequency performance.

Recent studies show that TFET seems to be a promising candidate for future low-power applications [12–16] and analog/RF applications [17–19]. However, due to the small effective tunneling area, the limited tunneling current becomes an inherent disadvantage in conventional P-I-N TFET, which leads to a low on-state operating current (*I*_ON_). In order to improve the TFET performance, many new structures have been proposed in recent years [20–25]. Benefiting from the recessed gate, L-shape tunnel field-effect transistor (LTFET) [23, 24] and U-shape tunnel field-effect transistor (UTFET) [25] have been proposed to obtain high *I*_ON_ with a compact device structure. However, there is still much room for improvement in LTFET and UTFET and needs to spend more effort to study the analog/RF performance of these devices.

In this paper, a T-shape gate dual-source tunnel field-effect transistor (TGTFET) with dual source is put forward and studied by TCAD simulation. The designed TGTFET can double the tunneling junction area compared with LTFET and UTFET. The gate overlap introduced by the designed T-shape gate can enhance the band-to-band tunneling rate (BBT rate). The simulation results show that the proposed TGTFET gains a higher *I*_ON_ (8.1 × 10^− 5^ A/μm at *V*_d_ = 1 V) than the LTFET and UTFET under the same condition. Both of the SS_min_ (at *V*_g_ = 0.1 V) and the SS_avg_ (0~0.5 V *V*_g_) of TGTFET are lower than 60 mV/dec (24.4 mV/dec and 51.5 mV/dec, respectively). TGTFET gains better input/output characteristic (*g*_m_ = 232 μS/μm, *g*_ds_ = 214 μS/μm) than the UTFET and LTFET. Moreover, the capacitance characteristics of TGTFET, UTFET, and LTFET are discussed in detail. Finally, TGTFET gains better analog/RF performance (*f*_T_ = 11.9 GHz and GBW = 2.3 GHz) compared to UTFET and LTFET. As a result, TGTFET with considerable good performance can be obtained.The structures of this paper are as follows: the “Methods” section includes the description of the structure and the parameters of TGTFET, LTFET [23, 24], and UTFET [25] as well as the TCAD simulation methods. The “Results and Discussion” section includes the description of the simulation results. In this section, the mechanism, characteristic, and analog/RF performance of TGTFET are studied and compared with the LTFET and UTFET. The influence of the device parameters on TGTFET is analyzed in detail too. The “Conclusions” section gives a conclusion of this paper.

## Methods

**Fig. 1:**
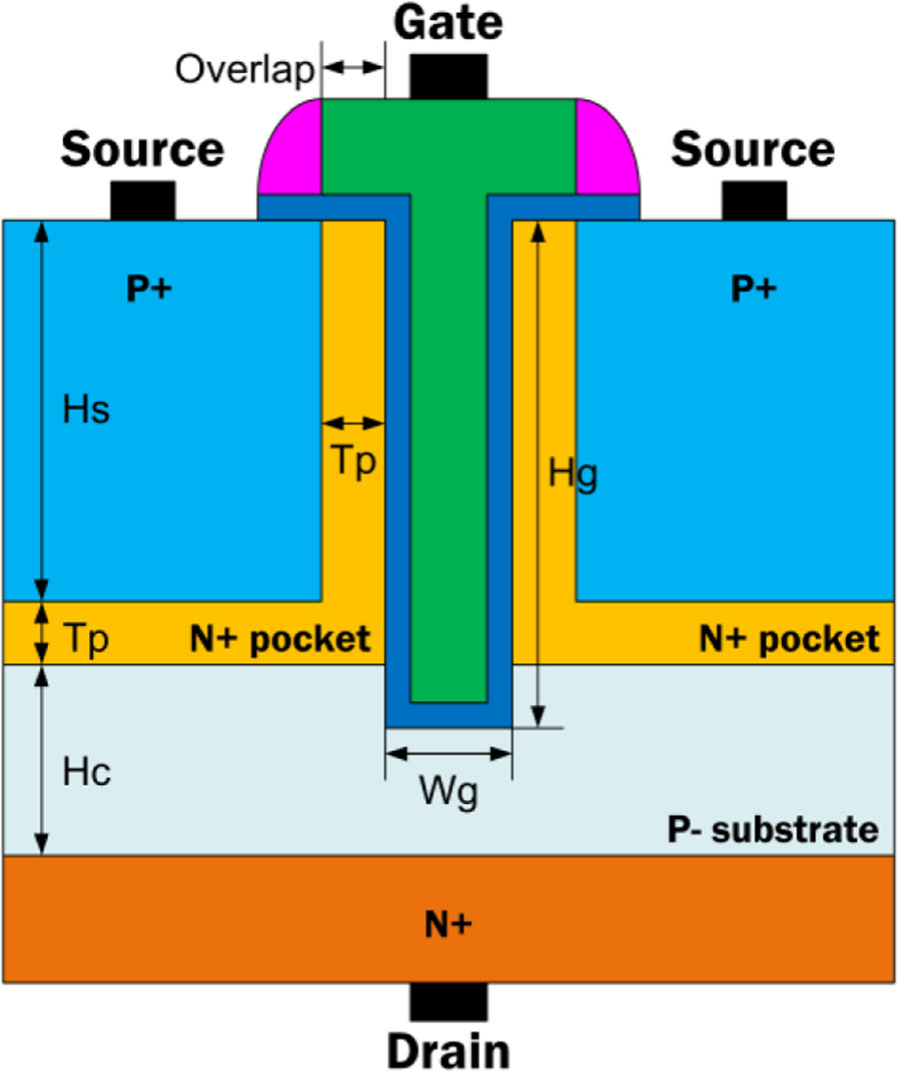
Schematic of the proposed T-shape gate dual-source tunnel field-effect transistor (TGTFET)

The structure of T-shape gate dual-source tunnel field-effect transistor (TGTFET) is illustrated in Fig. 1. The shape of the gate is similar to the alphabet letter “T” (green region). The dual-source regions are located on two sides of the gate (sapphire regions). Two n+ pockets (yellow regions) are inserted to increase the channel tunneling rate [20–22]. The n+ drain is placed in the bottom of the channel. Therefore, the T-shaped gate overlaps the n+ pockets in both the vertical and lateral directions. By this way, the electric field at the top of the tunneling junction can be increased. The electric field enhancement causes the energy band to bend more steeply. Finally, the electron tunneling rate is enhanced due to the corner electric field enhancement [26].

**Fig. 2:**
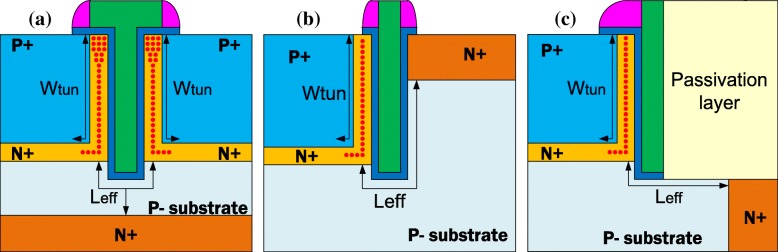
Comparison of **a** the proposed TGTFET, **b** UTFET, and **c** LTFET

Figure 2 shows the device structure of LTFET [23, 24], UTFET [25], and TGTFET. The gate overlap can help to enhance the tunneling efficiency of TGTFET. The dual-source regions in TGTFET can double the tunneling junction area compared with LTFET and UTFET.

Parameters of silicon-based TGTFET, UTFET, and LTFET used in simulations are as follows: Hs = 30 nm (height of the source region), Hg = 40 nm (height of the recessed gate), Wg = 6 nm (width of the gate region), Hc = 15 nm (height of the channel region), Tp = 5 nm (thickness of the n+ pocket), *ϕ* = 4*.*33 eV (gate work function), Tox = 2 nm (thickness of the HfO_2_ gate dielectric), *N*_S_ = 1 × 10^20^ cm^−3^ (p+ source doping concentration), *N*_D_ = 1 × 10^19^ cm^−3^ (n+ drain doping concentration), *N*_sub_ = 1 × 10^17^ cm^−3^ (p− substrate doping concentration), and *N*_P_ = 5 × 10^18^ cm^−3^ (n+ pocket doping concentration). The width coefficient in simulation is default to 1 μm.

Simulations of TGTFET, UTFET, and LTFET are carried out in Silvaco Atlas TCAD tools. Non-local BTBT model is introduced in this simulation to bring the energy band spatial variation into account, which can help to facilitate the accuracy of the BTBT tunneling process. Lombardi mobility model is considered to make the channel mobility more accurate (by considering the surface scattering including the transverse field and doping concentration). Fermi statistics and band gap narrowing model is taken into account to fit the effect of the highly doped regions. Shockley-Read-Hall recombination model is taken into account in this paper, too.

## Results and Discussion

### Device Mechanism and DC Characteristics with Different Parameters

**Fig. 3:**
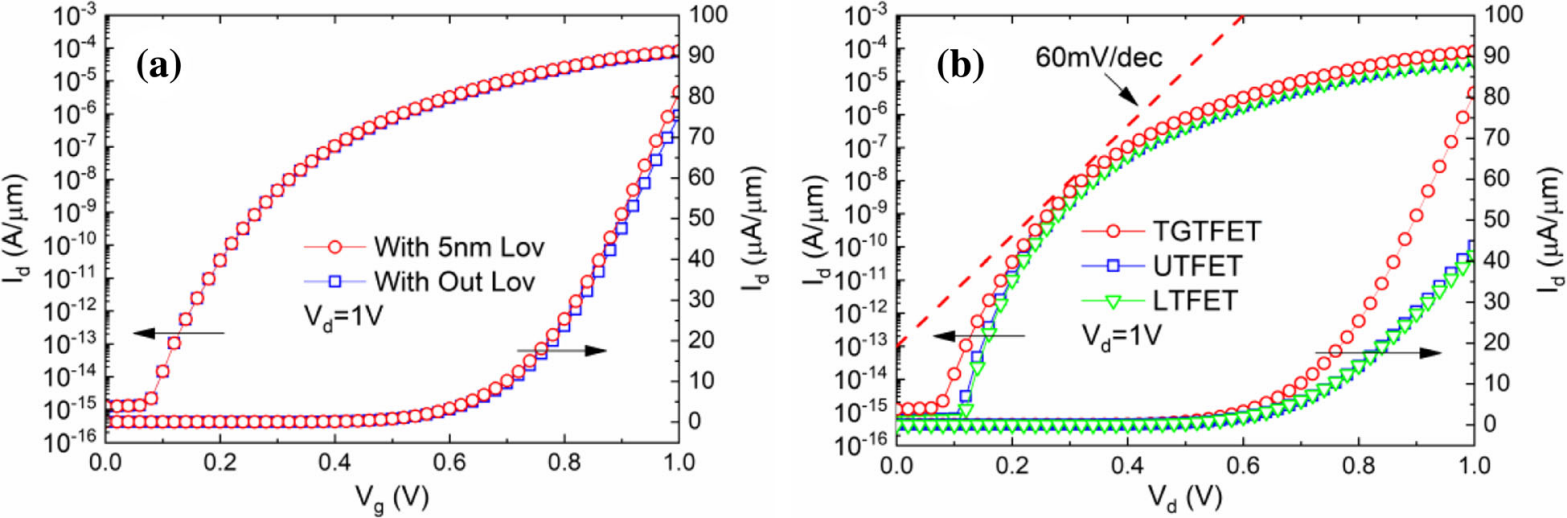
Simulated **a** transfer characteristics of TGTFET with/without gate overlap and **b** transfer characteristics of TGTFET, UTFET, and LTFET

Figure 3a shows the transfer characteristics of the TGTFET with and without the gate overlap. With the additional gate overlap, the *I*_ON_ increases from 7.5 × 10^−5^ to 8.1 × 10^−5^ A/*μ*m at *V*_g_ = *V*_d_ = 1 V. Figure 3b shows the transfer characteristic curves of TGTFET, UTFET, and LTFET. In order to make the comparison more accurate, the simulation models and geometric dimensions of these three devices are set to be identical. As a result, the TGTFET has about a twofold increase in *I*_ON_ compared with LTFET and UTFET, as shown in Fig. 3b. SS_min_ of TGTFET is 24.4 mV/dec at *V*_g_ = 0.1 V, and SS_avg_ is 51.5 mV/dec when 0 V < *V*_g_ < 0.5 V. The switching ratios (*I*_ON_/*I*_OFF_) are 6.7 × 10^10^ at *V*_g_ = *V*_d_ = 1 V and 6.5 × 10^8^ at *V*_g_ = *V*_d_ = 0.5 V.

**Fig. 4:**
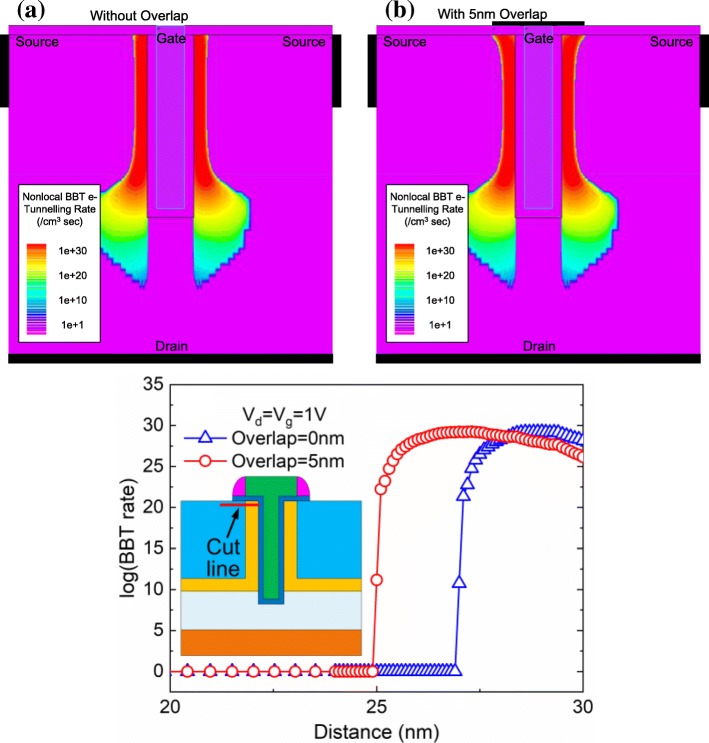
Simulated BBT electron tunneling rate diagrams of **a** device without gate overlap, **b** device with 5-nm gate overlap, and **c** the BBT electron tunneling rate of two devices, at 1 nm below the device surface; *V*_g_ = *V*_d_ = 1 V

Figure 4a, b shows the BBT rate of TGTFET with and without a 5-nm gate overlap. From Fig. 4c, we can clearly see that the device with a 5-nm gate overlap has a wider electron tunneling area under the device surface, which can lead to the *I*_ON_ increasing.

**Fig. 5:**
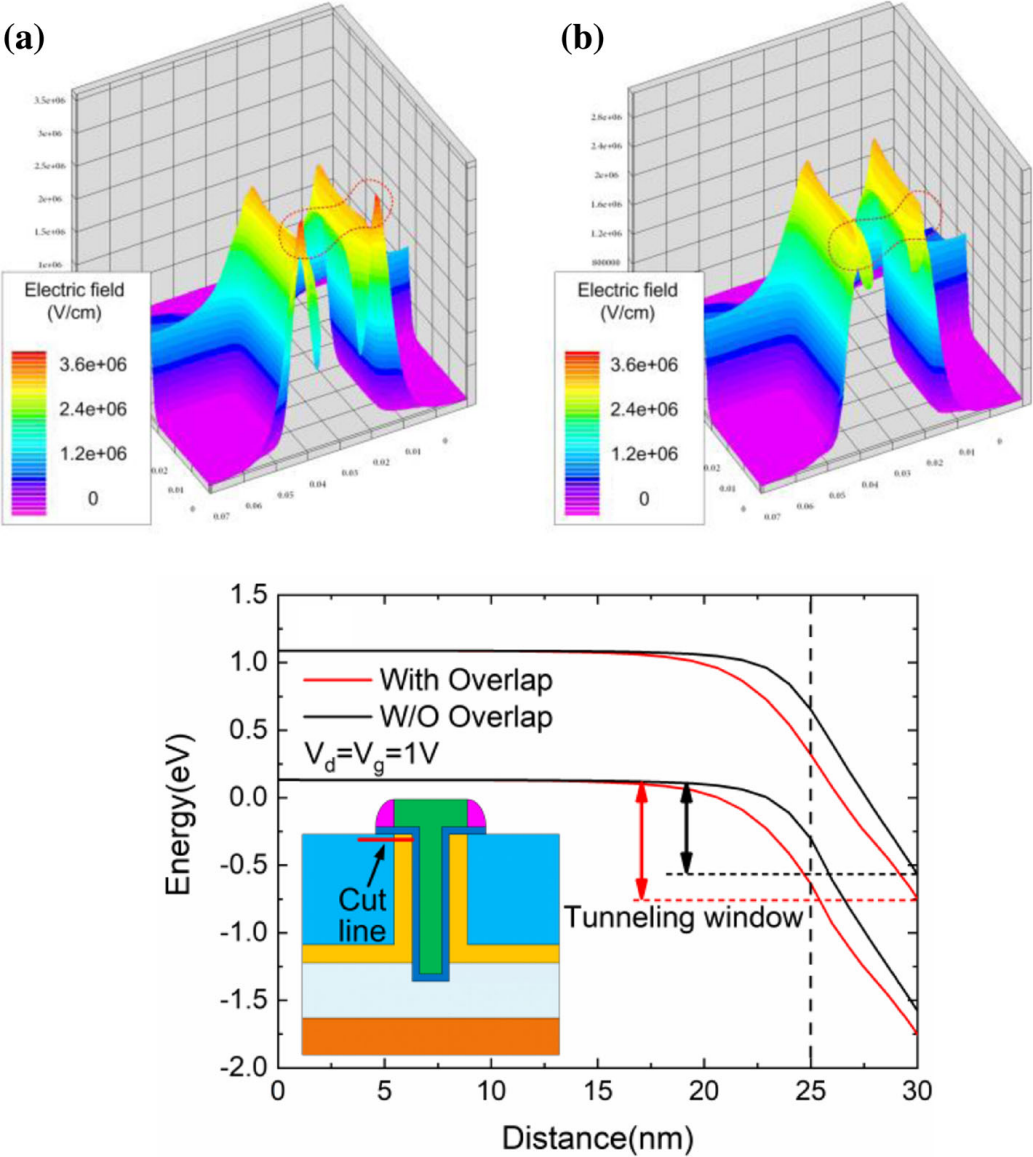
3D schematic diagram of electric fields of the device **a** with overlap and **b** without overlap; simulated **c** energy band diagrams from source to pocket region (1 nm below the oxide interface)

Figure 5a, b shows the 3D diagram of electric fields of TGTFET with and without gate overlap. Two electric field peaks appear in TGTFET with a 5-nm gate overlap, as shown in the dashed circle in Fig. 5a. No electric field peak appears in Fig. 5b attributed to the absences of the gate overlap. Figure 5c shows the energy band structure under the surface of the device. The inset in Fig. 5c shows the cut line location. With the gate overlap, a larger tunneling window can be obtained. Thus, a higher BBT rate and *I*_ON_ can be achieved.

**Fig. 6:**
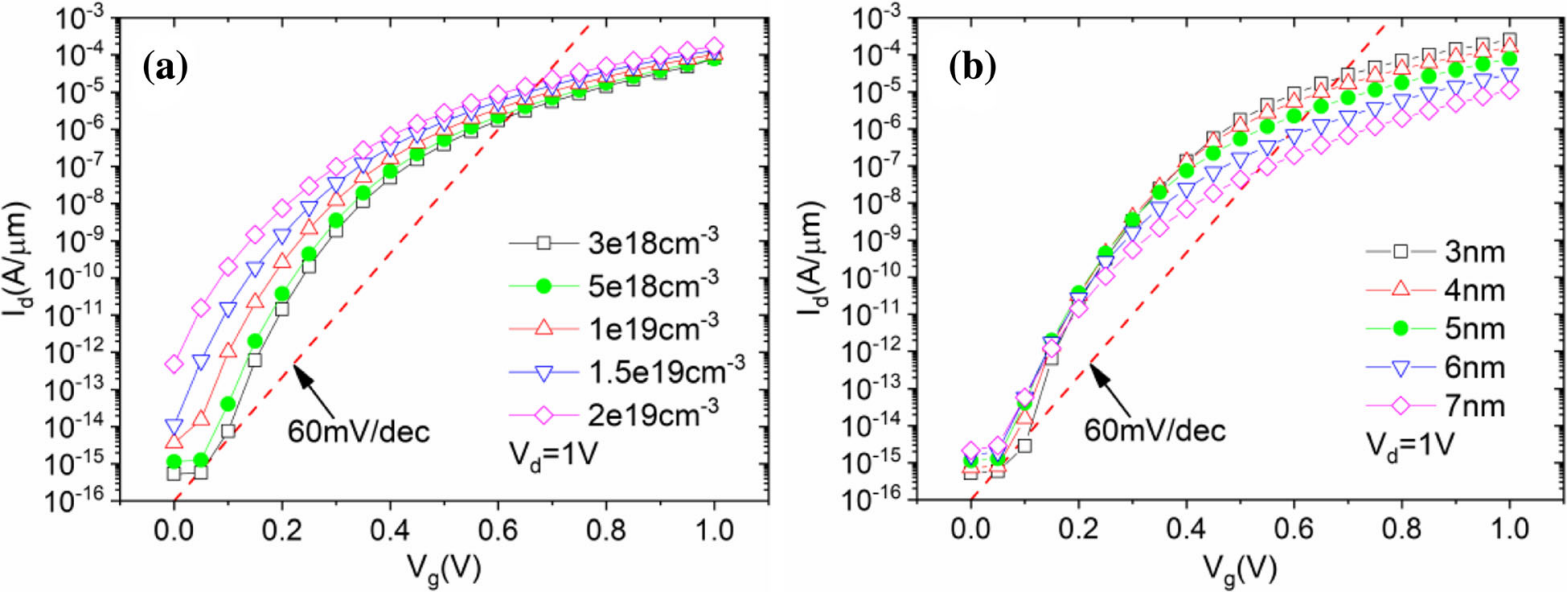
Simulated drain currents with different n+ pocket **a** concentrations and **b** thicknesses at *V*_d_ = 1 V

Figure 6 shows the effects of n+ pocket on the performance of the TGTFET. The *I*_OFF_ increases rapidly with the increasing of the n+ pocket doping concentration, as shown in Fig. 6a. The lower SS and greater *I*_ON_ can be obtained by decreasing the thickness of n+ pocket (Tp) from 7 to 3 nm when *N*_P_ = 5 × 10^18^ cm^−3^, as shown in Fig. 6b. At the same time, no significant subthreshold current is noted in Fig. 6b. It can be confirmed from Fig. 6a that a relatively low doping concentration of n+ pocket will help to suppress the subthreshold current.

**Fig. 7:**
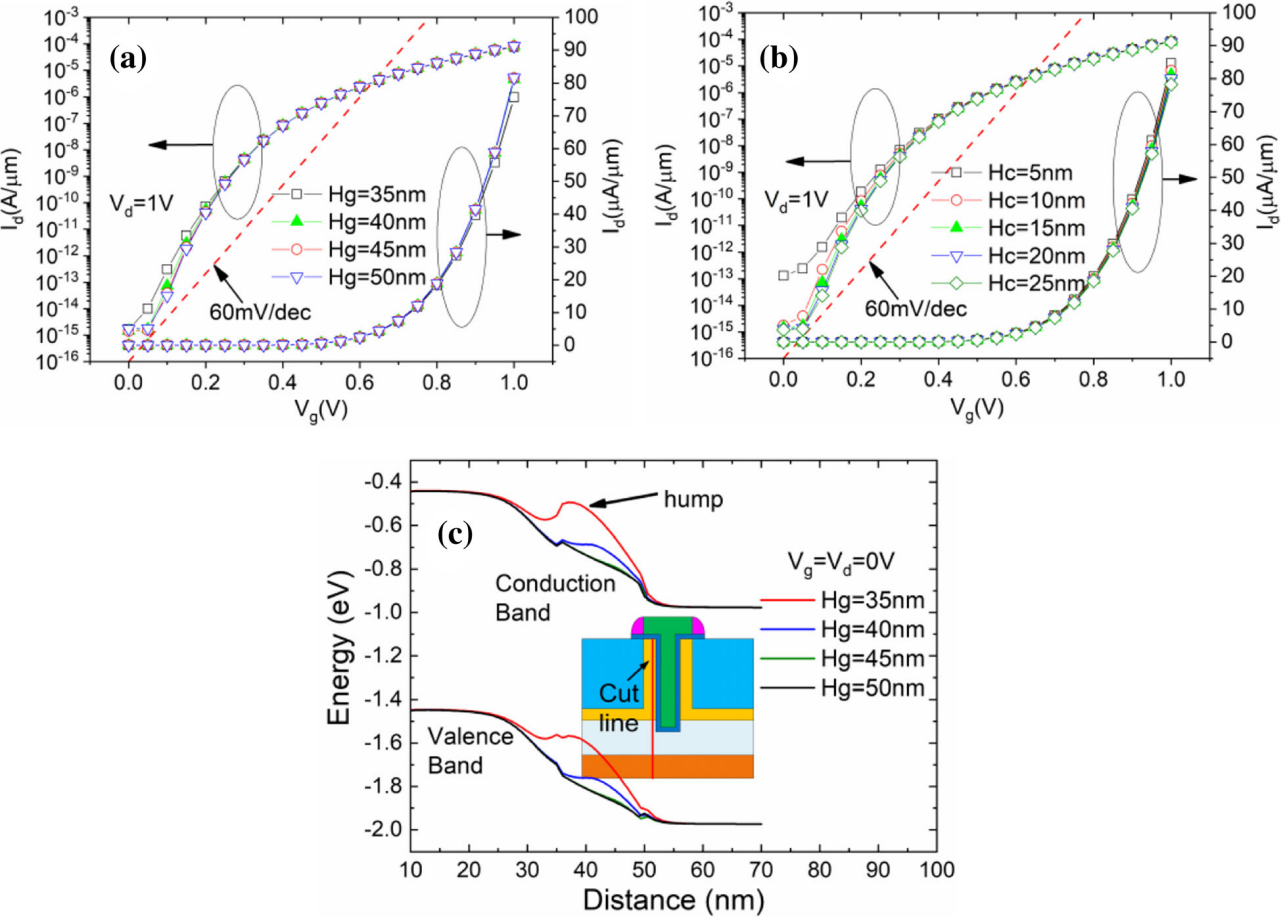
Simulated transfer characteristics of TGTFET with **a** different Hg, **b** different Hc, and **c** the conduction band hump on the current path

**Fig. 8:**
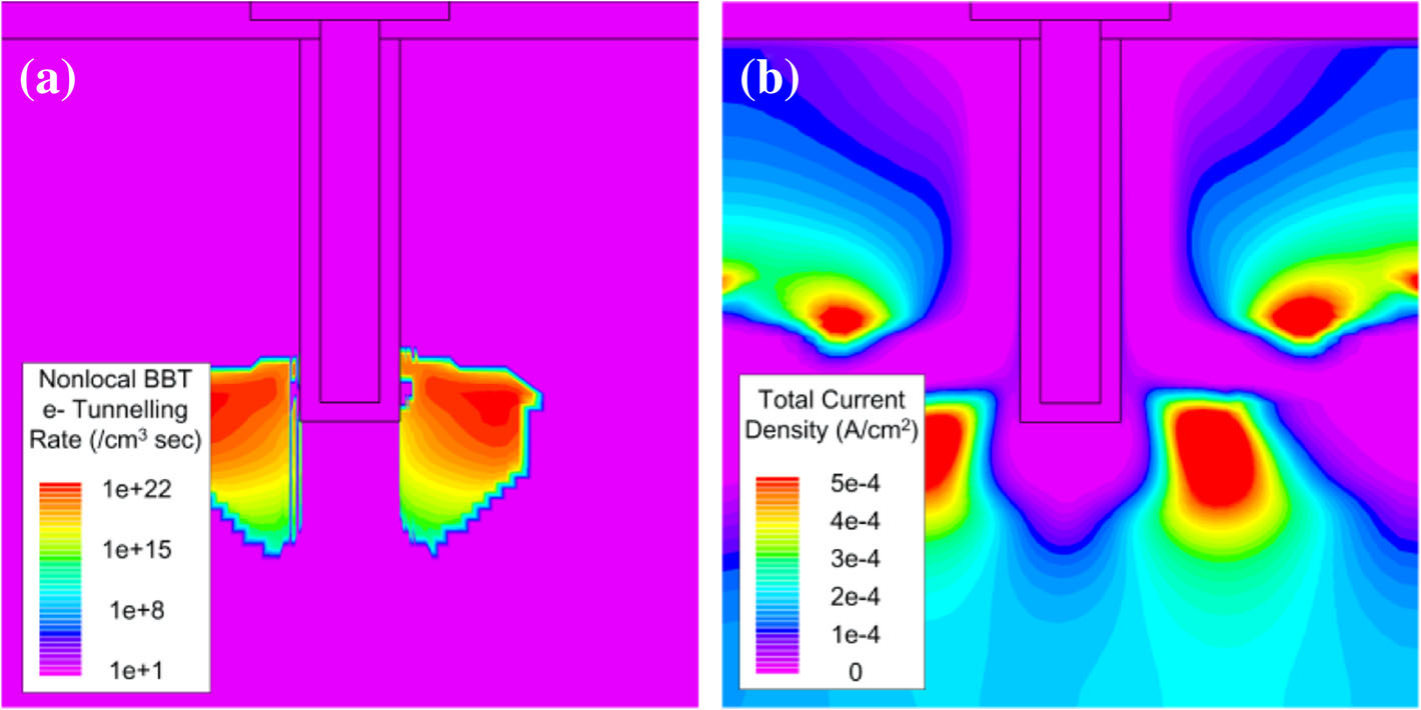
Simulated diagrams of off-state **a** BTBT electron tunneling rate and **b** current density when Hc = 5 nm

The impact of the gate height (Hg) and channel thickness (Hc) is shown in Fig. 7a, b, separately. A small *I*_ON_ and SS improvement appears when Hg is increasing. Because when Hg = 35 nm, there is an obvious energy band hump on the on-state current path, becoming a certain obstacle to the lucky electrons (electrons which passed the tunneling junction), as shown in Fig. 7c, which can result in *I*_on_ decrease. When Hg increases, the energy band hump is weakened, which cause the *I*_ON_ and SS improvement. A slight *I*_ON_ improvement is obtained with Hc decreasing, as shown in Fig. 7b. However, severe degradation on subthreshold characteristic can be observed when Hc decreases to 5 nm. This can be explained by the increasing subthreshold tunneling current at the corner of the n+ pocket, as shown in Fig. 8. Figure 8a shows the obvious off-state band-to-band tunneling phenomenon when Hc = 5 nm while Fig. 8b shows the *I*_OFF_ current density when Hc = 5 nm.

**Fig. 9:**
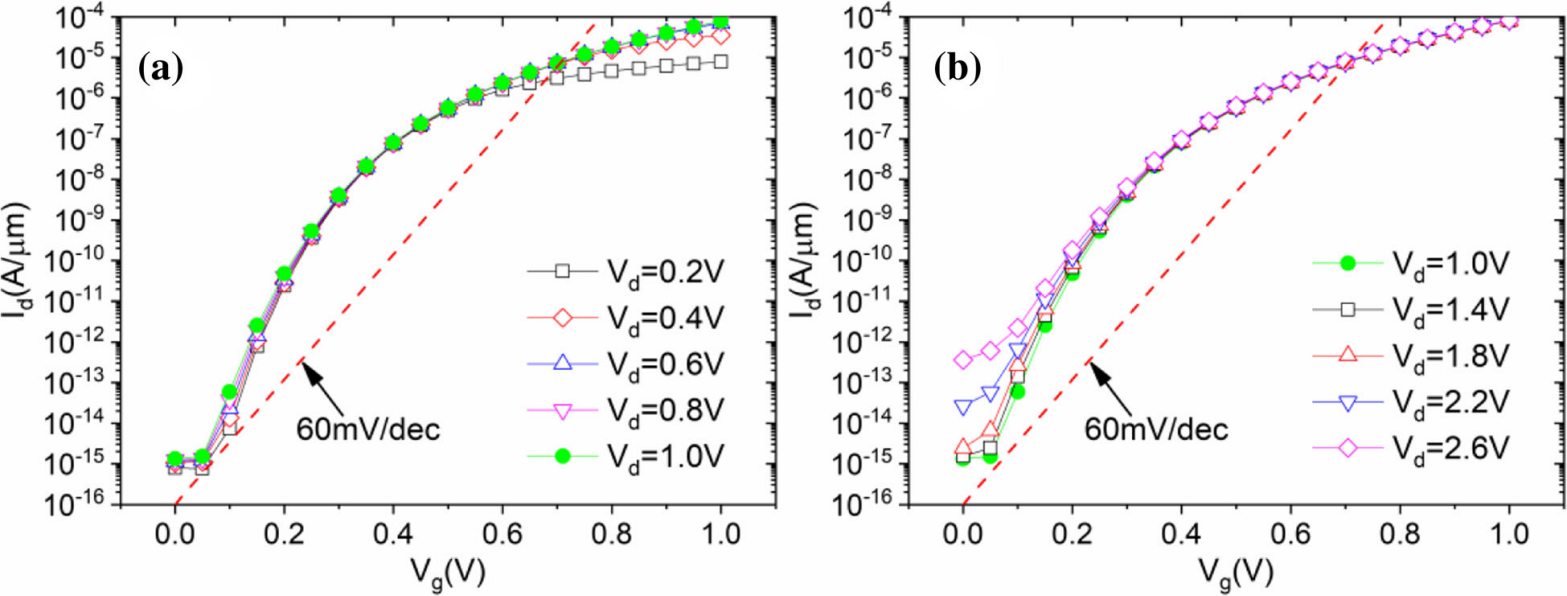
Simulated drain currents for **a** *V*_d_ ≤ 1 V and **b** *V*_d_ ≥ 1 V

As shown in Fig. 9, the influence of drain to source voltage (*V*_d_) is also taken into account in this paper. For *V*_d_ < 0.6 V, *I*_ON_ increases obviously with the increasing *V*_d_, as shown in Fig. 9a. This is explained by the fact that the potential of the p-channel is slowly growing in response to the increasing *V*_d_ and results in the decreasing resistance of p-channel. For *V*_d_ > 1.8 V, shown in Fig. 9b, the *I*_ON_ almost does not increase with the increasing *V*_d_, but *I*_OFF_ increases considerably. This is because of the subthreshold tunneling current at the corner of the n+ pocket increasing rapidly with the increasing *V*_d_. Finally, for 0.6 V < *V*_d_ < 1.8 V, TGTFET exhibits good and stable performance. As a result, TGTFET is robust to drain-induced barrier lowering (DIBL) and exhibits a good and stable performance in a larger applied voltage dynamic range.

### Analog/RF Performance of TGTFET, UTFET, and LTFET

**Fig. 10:**
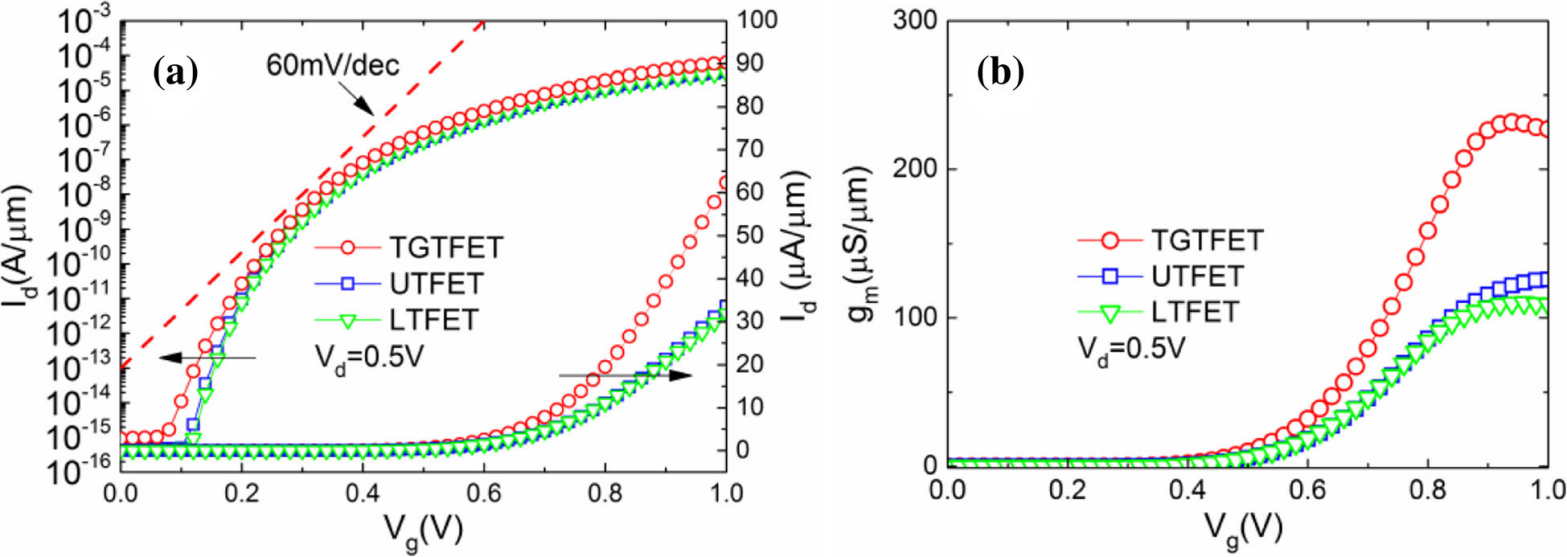
**a** Transfer characteristics and **b** transconductance curves of TGTFET, UTFET, and LTFET at *V*_d_ = 0.5 V

Figure 10 shows the transfer characteristics and transconductance curves of TGTFET, UTFET, and LTFET at *V*_d_ = 0.5 V. The transconductance (*g*_m_) can be obtained from the first derivative of the transfer characteristic curve, as shown in Eq. (1) [27–29]:

1 $$ {g}_{\mathrm{m}}={dI}_{\mathrm{d}s}/{dV}_{\mathrm{gs}} $$

As a result, the maximum transconductance of TGTFET (232 μS/μm) is about two times larger than that of UTFET (120 μS/μm) and LTFET (110 μS/μm), as shown in Fig. 10. This is benefited from the current gain contributed by dual source and gate overlap.

**Fig. 11:**
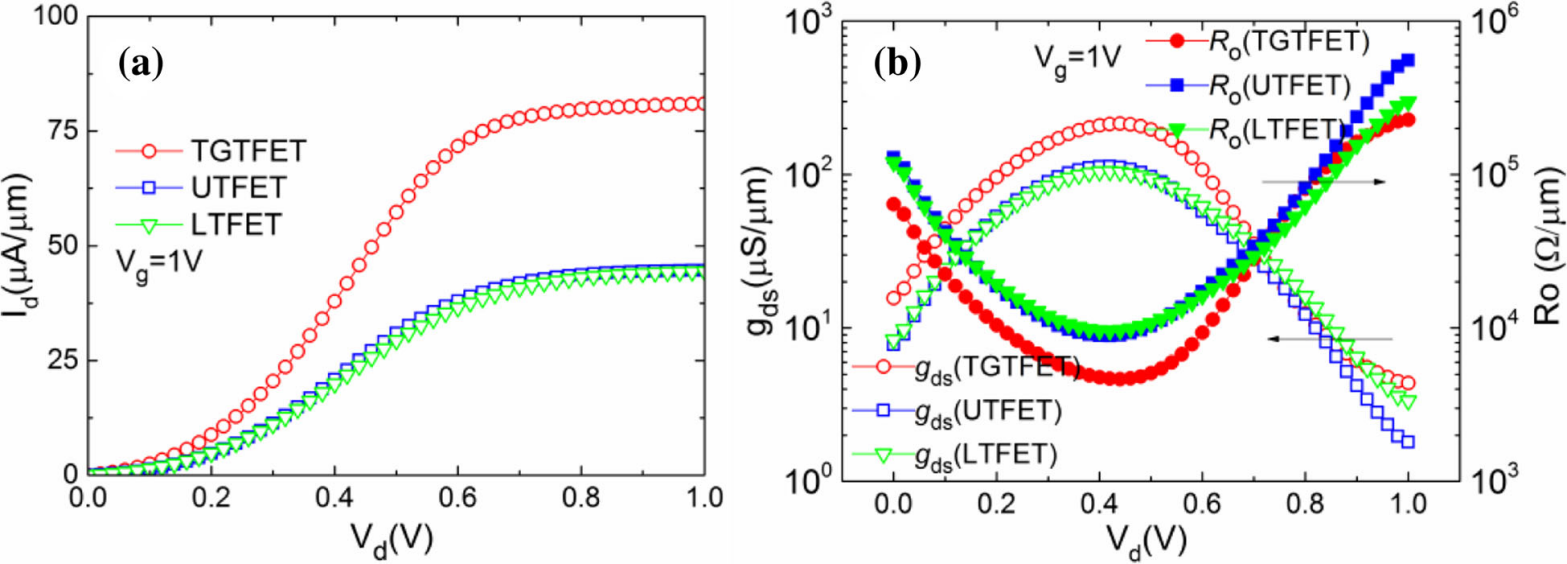
**a** Output characteristics, **b** output conductance (*g*_ds_), and **c** output impedance (*R*_o_) curves of the TGTFET, UTFET, and LTFET

Figure 11 shows the output characteristics, output conductance (*g*_ds_), and output impedance (*R*_o_) curves of the TGTFET, UTFET, and LTFET. As shown in Fig. 11a, it can be clearly seen that the output current of the device increases with the increase of *V*_d_, but when *V*_d_ reaches above 0.6 V, the output current tends to saturate. Through observation, it is easy to find that the output current of TGTFET is two times larger than that of UTFET and LTFET. Figure 11b shows the output conductance (*g*_ds_) and output impedance (*R*_o_) curves of the TGTFET, UTFET, and LTFET. The *g*_ds_ can be obtained through the derivation of the output current, as shown in Eq. (2) [27, 29] while *R*_o_ can be expressed as the reciprocal of the output conductance.

2 $$ {g}_{\mathrm{ds}}={dI}_{\mathrm{ds}}/{dV}_{\mathrm{ds}} $$

Due to the advantages on output current, TGTFET gains the highest *g*_ds_ and the minimum *R*_o_ of these three devices. Under 1-V gate bias condition, TGTFET obtained the maximum *g*_ds_ of 214 μS/μm and the minimum *R*_o_ of 4.6 kΩ/μm under 0.45 V *V*_d_. Under the same gate bias condition, UTFET and LTFET obtained the maximum *g*_ds_ of 113 μS/μm and 105 μS/μm and the minimum *R*_o_ of 9.0 kΩ/μm and 9.6 kΩ/μm under 0.4 V *V*_d_.

**Fig. 12:**
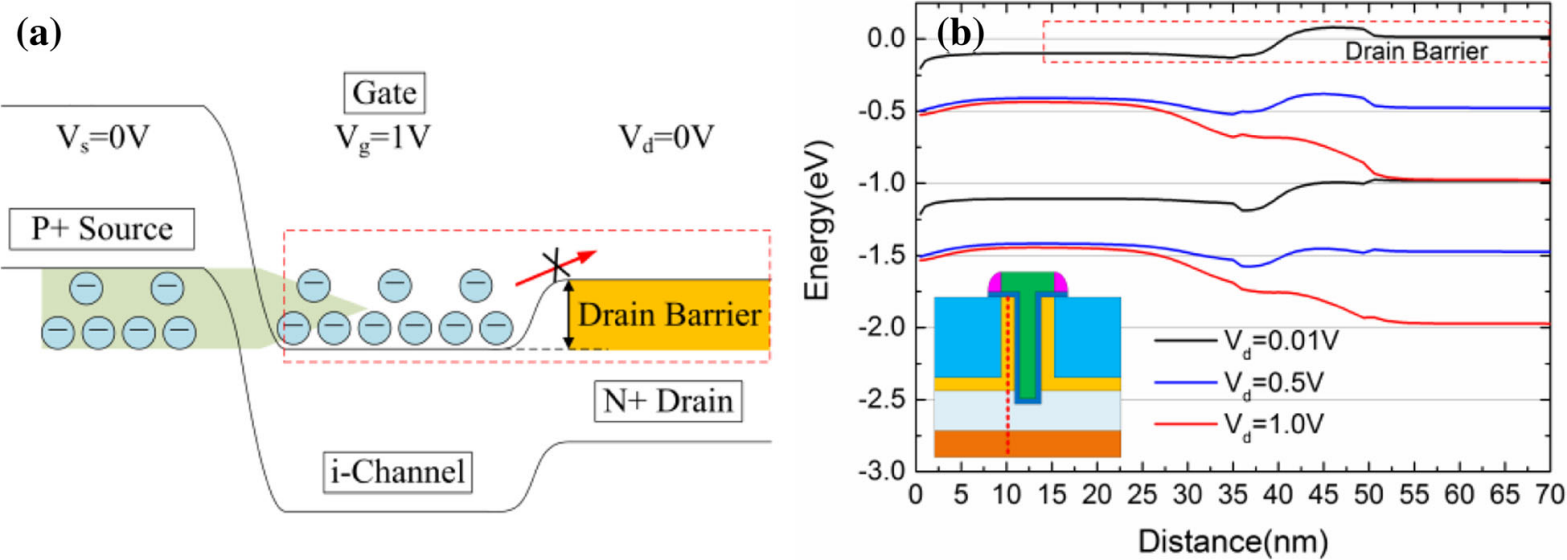
**a** Schematic diagram of the energy band at *V*_d_ = 0 V and *V*_g_ = 1 V. **b** Simulation results of the energy band diagram at different biases of *V*_d_

Moreover, in Fig. 11, it is not difficult to find out that the linear region of the device output characteristics shows certain nonlinearity. As shown in Fig. 11a, *R*_o_ decreases first and then increases with the increasing *V*_d_. Some research groups give the corresponding physical process about this phenomenon [7, 30] but there are still some problems that have not been explained clearly. As we know, *R*_o_ is determined by the resistance of channel region and tunneling junction. When *V*_d_ < 0.4 V, *R*_o_ decreases with the increasing *V*_d_. Consider the following situations, when *V*_d_ = 0 V and *V*_g_ = 1 V, none of the lucky electrons can be swept to the drain side, and almost all the electrons are trapped in the channel region by a relatively high drain barrier, as shown in the red dotted line frame in Fig. 12a, b. When 0 V < *V*_d_ < 0.4 V, with the increasing of *V*_d_, the drain barrier becomes weaker (as shown in Fig. 12b). Thus, the electrons trapped in the channel region can pass through the drain barrier and then be collected by drain. This is a thermal excitation process of electrons from channel to drain. Finally, as the tunneling junction has been completely turned on (when *V*_g_ = 1 V), the tunneling current is always in a state of excess and the resistance introduced by tunneling junction can be ignored. At this time, *R*_o_ is determined by the channel resistance and *R*_o_ is decided by the electron thermal excitation process across the drain barrier. Thus, *R*_o_ decreases with the increasing of *V*_d_. When *V*_d_ > 0.6 V, these three devices gradually enter the saturation area and *R*_o_ becomes larger. This is because when *V*_d_ is large, almost all the electrons through the tunneling junction are swept to the drain side by the relatively high electric field. The tunneling current becomes the limit of the drain current. In this condition, *R*_o_ is mainly determined by the tunneling junction. However, the tunneling efficiency cannot increase significantly while *V*_d_ is increasing. *V*_d_ has a small effect on the energy band structure of the tunneling junction (n+ pocket side), as shown in Fig. 12b. As a result, the tunneling current cannot increase obviously, and there is almost no *I*_ON_ increase with the continually increasing *V*_d_ (when *V*_d_ > 0.6 V), which means an impedance increases. Moreover, when 0.4 V < *V*_d_ < 0.6 V, *R*_o_ is determined by both the channel resistance and tunneling junction.

It can be obtained from the above analysis that the *R*_o_ of TFET is influenced by both the tunneling process and the channel electron thermal excitation process. The main physical mechanisms can dominate *R*_o_ shifts with *V*_d_ variation. Finally, the *R*_o_ decreases first and then increases, thus causing the nonlinearity of the output characteristics. Incidentally, through the observation of Fig. 11b, it is easy to find that the output impedance of TGTFET is much smaller than that of the UTFET and LTFET. This is due to the better tunneling efficiency benefit from the dual-source and the lateral gate overlap structure of TGTFET.

**Fig. 13:**
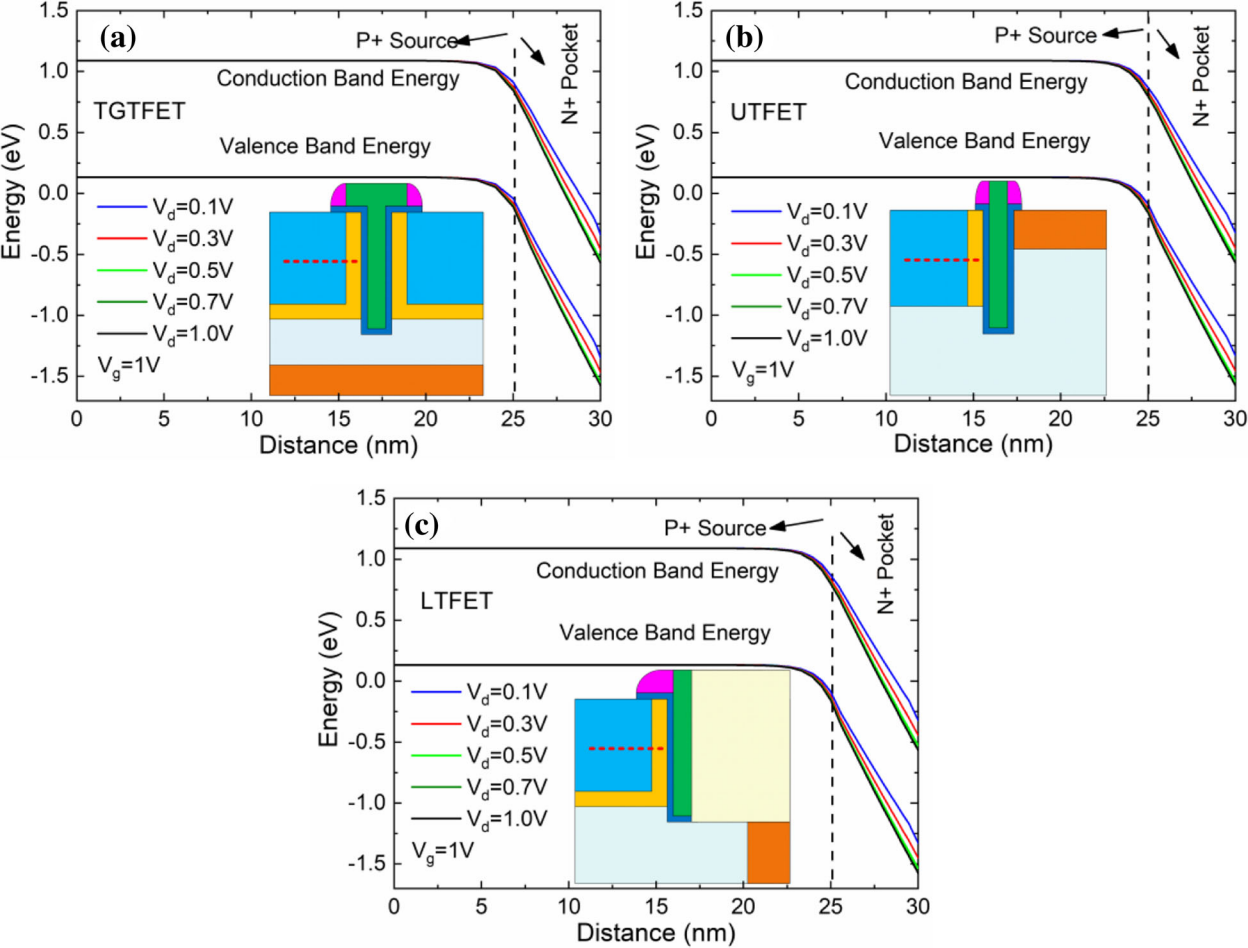
The energy band structure of **a** TGTFET, **b** UTFET, and **c** LTFET at *V*_g_ = 1

Figure 13 shows the energy band structure of TGTFET, UTFET, and LTFET with different applied voltages. The red dotted lines in the inset represent the position to draw the energy band (which is 15 nm below the surface, just at the 1/2 height of the source region). It can be seen that with a *V*_d_ increase from 0.1 to 0.5 V, the band structure of TGTFET, UTFET, and LTFET has an obvious trend of bending. This is because the drain voltage can pull down the electric potential of the tunneling junction near the drain side. This indicates that, for TGTFET, UTFET, and LTFET, the increase of *V*_d_ from 0.1 to 0.5 V is beneficial to tunneling efficiency. However, when *V*_d_ > 0.5 V, the change of the energy band with *V*_d_ increase is not worth mentioning. This is consistent with the analysis results in Fig. 12b.

**Fig. 14:**
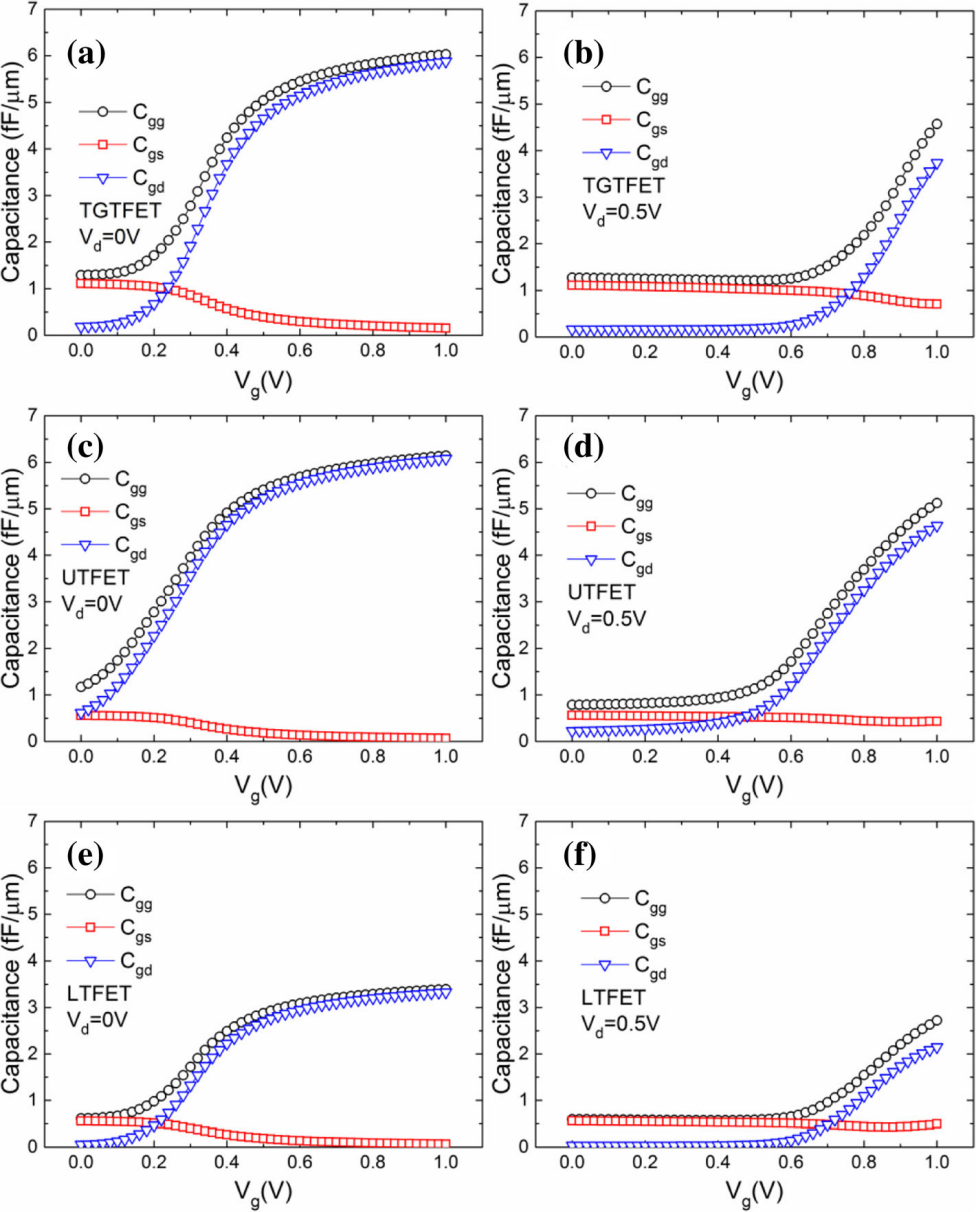
Capacitance of TGTFET versus *V*_g_ under **a** *V*_d_ = 0 V and **b** *V*_d_ = 0.5 V. Capacitance of UTFET versus *V*_g_ under **c** *V*_d_ = 0 V and **d** *V*_d_ = 0.5 V. Capacitance of LTFET versus *V*_g_ under **e** *V*_d_ = 0 V and **f** *V*_d_ = 0.5 V

As we know, the gate capacitance (*C*_gg_) of the device can greatly affect the frequency characteristics of the integrated circuits. For TGTFET, UTFET, and LTFET, *C*_gg_ generally consists of *C*_gs_ (capacitance of gate to source) and *C*_gd_ (gate to drain capacitance). Therefore, the characteristic of *C*_gg_, *C*_gs_, and *C*_gd_ is of great significance to evaluate the frequency characteristics and analog application ability of devices. Especially for TFET, the capacitance characteristics are quite different from MOSFET. Because of the existence of the tunneling junction at the source area, TFET usually has a small *C*_gs_ [1, 11]. Therefore, the *C*_gg_ of TFET is mainly determined by *C*_gd_. Figure 14 shows the capacitance of TGTFET, UTFET, and LTFET versus *V*_g_ under *V*_d_ = 0.5 V and *V*_d_ = 0 V, separately.

Through the observation of Fig. 14a, b, it is easy to find that the *C*_gs_ of TGTFET under 1-V gate voltage is 0.15 fF/μm at *V*_d_ = 0 V and 0.7 fF/μm at *V*_d_ = 0.5 V, which is far more smaller than that of the *C*_gd_ (5.8 fF/μm at *V*_d_ = 0 V and 3.7 fF/μm at *V*_d_ = 0.5 V). Thus, the *C*_gg_ of TGTFET is mainly determined by *C*_gd_. When *V*_d_ = 0 V, *C*_gg_ and *C*_gd_ increase rapidly with the increasing *V*_g_, as shown in Fig. 14a. This is because with the increase of *V*_g_, electrons are aggregated to the gate interface in the device channel, which makes the capacitance rise rapidly. When *V*_d_ = 0.5 V, *C*_gd_ does not increase obviously until *V*_g_ is increased to more than 0.6 V, as shown in Fig. 14b. This is because when *V*_g_ is low, only few lucky electrons can pass through the tunneling junction and go into the channel. Some of these lucky electrons will be participating in the recombination process, and most of the others will be rapidly collected by drain due to the 0.5-V drain voltage. Therefore, it is very difficult for these lucky electrons to stay in the device channel. However, with the *V*_g_ increase, the number of lucky electrons increases rapidly. At this moment, neither of the drain collection nor of the electron-hole recombination process can rapidly deplete these lucky electrons. Thus, the electron concentration in the channel increases and the capacitance rises rapidly. As a result, the capacitance characteristic curve tends to shift right while *V*_d_ increases, as shown in Fig. 14a, b. The above analysis and phenomena are also applicable to UTFET and LTFET, as shown in Fig. 14c–f. In addition, the gate capacitance of UTFET at 0 V and 0.5 V *V*_d_ reached 6.2 fF/μm and 5.1 fF/μm, respectively, and that of the LTFET reached 3.4 fF/μm and 2.7 fF/μm, respectively.

**Fig. 15:**
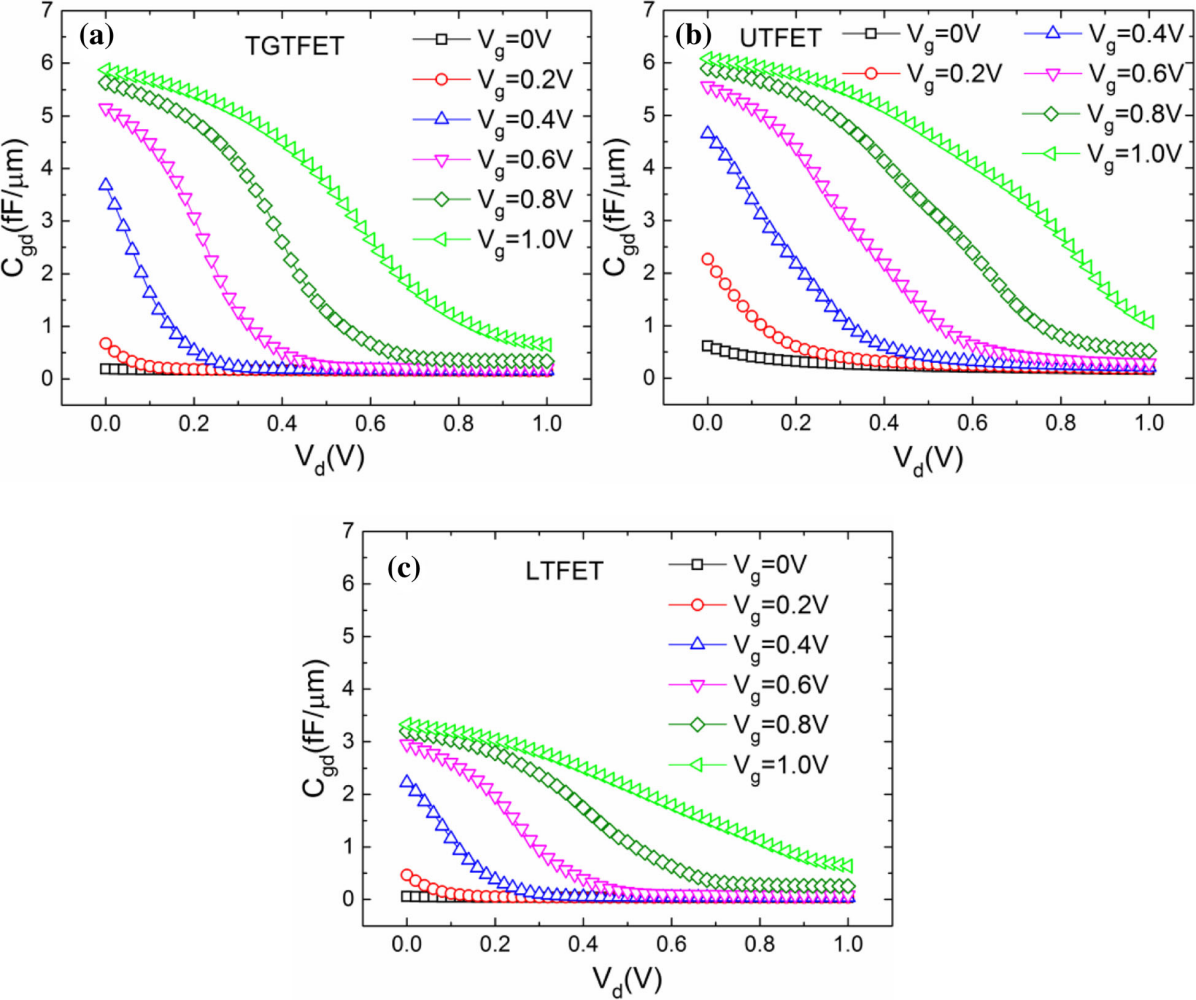
*C*_gd_ characteristics of **a** TGTFET, **b** UTFET, and **c** LTFET versus *V*_d_ under different *V*_g_

Since there is no direct overlap between the LTFET’s gate and drain, and the distance between the gate and drain is relatively far, LTFET has the best capacitance characteristics and the smallest *C*_gg_. In contrast, there is a direct overlap between the UTFET’s gate and drain. Therefore, electrons near the drain side are more easily controlled by gate, thus resulting in a large *C*_gg_ of UTFET. For TGTFET, although the distance between the gate and drain is close, but there is a lightly doped channel region which can isolate the gate and drain. Thus, the capacitance of TGTFET is better than that of the UTFET, but slightly inferior to LTFET. Figure 15 shows the *C*_gd_ characteristics of TGTFET, UTFET, and LTFET versus *V*_d_ under different *V*_g_. From the observation of Fig. 15a–v, it is not difficult to find that the *C*_gd_ characteristics of these three devices are similar. That is, for a fixed *V*_g_, *C*_gd_ decreases with the increase of the *V*_d_. On the other hand, for a fixed *V*_d_, *C*_gd_ increases with the increase of *V*_g_.

As we know, both of the cut-off frequency (*f*_T_) and gain bandwidth (GBW) are the evaluation criteria for evaluating the frequency characteristics of devices. *f*_T_ depends on the ratio of *g*_m_ to *C*_gg_, as shown in Eq. (3) [30, 31]. For a certain DC gain that equals 10, GBW can be expressed by the ratio of *g*_m_ to *C*_gd_, as shown in Eq. (4) [17]:

3 $$ {f}_T=\frac{g_{\mathrm{m}}}{2\pi {C}_{\mathrm{gs}}\sqrt{1+2{C}_{\mathrm{gd}}/{C}_{\mathrm{gs}}}}\approx \frac{g_{\mathrm{m}}}{2\pi \left({C}_{\mathrm{gs}}+{C}_{\mathrm{gd}}\right)}=\frac{g_{\mathrm{m}}}{2\pi {C}_{\mathrm{gg}}} $$

4 $$ \mathrm{GWB}={g}_{\mathrm{m}}/2\pi 10{C}_{\mathrm{gd}} $$

**Fig. 16:**
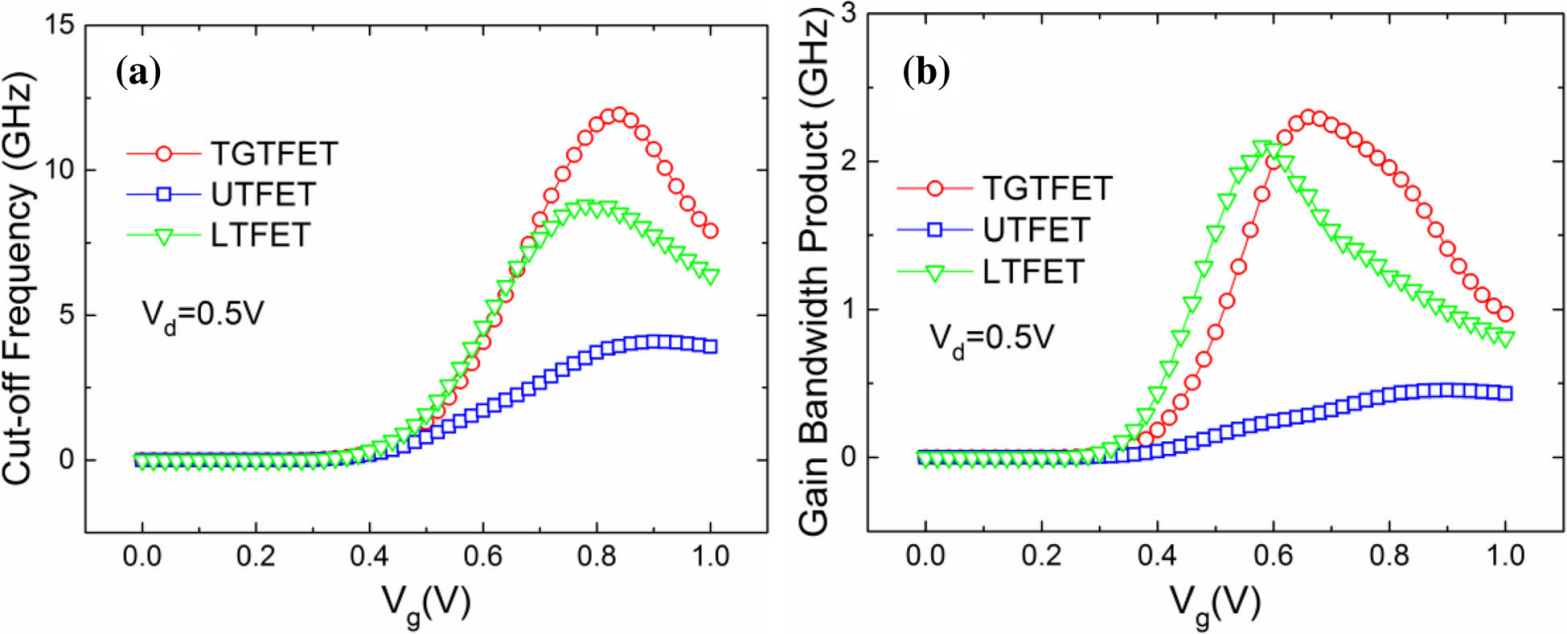
The characteristic curves of **a** *f*_T_ and **b** GBW of TGTFET, UTFET, and LTFET versus *V*_g_ at *V*_d_ = 0.5 V

Figure 16 shows the characteristic curves of the *f*_T_ and GBW of TGTFET, UTFET, and LTFET. Benefiting from structural advantages, such as dual-source and lateral gate overlap introduced by the T-shaped gate, TGTFET obtains the most outstanding frequency characteristics compared with UTFET and LTFET. Under the condition of *V*_d_ = 0.5 V, the *f*_T_ and GBW of TGTFET reached the maximum values of 11.9 GHz and 2.3 GHz, respectively. Benefiting from the long distance between gate and drain and without gate/drain overlap, LTFET obtains a small *C*_gg_ and good frequency characteristics. The *f*_T_ and GBW of LTFET reach the 8.7 GHz and 2.1 GHz, separately. The capacitance characteristics of UTFET are inferior compared with that of TGTFET and LTFET. This is because the direct gate/drain overlaps. As a result, the maximum value of *f*_T_ and GBW of UTFET can only reach 4.1 GHz and 0.5 GHz separately.

## Conclusions

In this paper, a T-shape gate dual-source tunnel field-effect transistor (TGTFET) with good performance is proposed and investigated. The structure, mechanism, and the influence of device parameter on the characteristic of TGTFET are discussed. In addition, the characteristics of TGTFET, UTFET, and LTFET are discussed and compared in this paper. The dual-source regions are introduced to double the area of the tunneling junction. The gate overlap and the n+ pockets can obviously enhance the tunneling efficiency of the tunneling junction in TGTFET. Finally, the TGTFET with impressive characteristics (*I*_ON_ = 8.1 × 10^−5^ A/μm, *I*_ON_/*I*_OFF_ = 6.7 × 10^10^ and SS_min_ = 24.4 mV/dec) is obtained. At the same time, TGTFET is robust to DIBL, which means TGTFET can exhibit a good and stable performance in a larger applied voltage dynamic range. Furthermore, the analog/RF performance of TGTFET is studied and compared with UTFET and LTFET. The key parameter such as input/output characteristics, capacitance characteristics, GBW, and *f*_T_ are analyzed. Benefiting from the no direct overlap between the gate and drain, TGTFET obtains a relatively small *C*_gd_ and *C*_gg_. Finally, TGTFET with remarkable frequency characteristics (*f*_T_ = 11.9 GHz and GBW = 2.3 GHz) is obtained. As a conclusion, it is expected that TGTFET can be one of the promising alternatives for the next generation of device in low-power and analog/RF applications.

## Abbreviations

*C*_gd_: Gate to drain capacitance
*C*_gs_: Gate to source capacitance
*f*_T_: Cut-off frequency
GBW: Gain bandwidth
*g*_ds_: Output conductance
*g*_m_: Transconductance
Hc: Height of the channel layer
Hg: Height of the gate electrode
Hs: Height of the source layer
LTFET: L-shape gate tunnel field-effect transistor
*N*_D_: Doping concentration of n+ drain
*N*_P_: Doping concentration of n+ pocket
*N*_S_: Doping concentration of p+ source
*N*_sub_: Doping concentration of p− substrate
*R*_o_: Output impedance
TGTFET: T-shape gate dual-source tunnel field-effect transistor
Tox: Thickness of the HfO_2_ gate dielectric
Tp: Thickness of n+ pocket
UTFET: U-shape gate tunnel field-effect transistor
*V*_d_: Drain to source voltage
*V*_g_: Gate to source voltage
Wg: Width of the gate electrode
